# Comparison of effectiveness between transthoracic echocardiography and X-ray guided closure of patent foramen ovale: A retrospective analysis

**DOI:** 10.12669/pjms.40.5.8994

**Published:** 2024

**Authors:** Haijing Song, Yanzhi Yao

**Affiliations:** 1 Haijing Song, Department of Ultrasound, The First People’s Hospital of Yongkang City, 599 Jinshan West Road, Yongkang City, Zhejiang Province 321300, P.R. China; 2 Yanzhi Yao, Department of Ultrasound, The First People’s Hospital of Yongkang City, 599 Jinshan West Road, Yongkang City, Zhejiang Province 321300, P.R. China

**Keywords:** Patent foramen ovale, Transthoracic echocardiography, Closure, X-ray

## Abstract

**Objective:**

To compare the effectiveness of transthoracic echocardiography (TTE) and X-ray guided closure of patent foramen ovale (PFO).

**Methods:**

In this retrospective study, clinical data from 90 patients who underwent PFO occlusion surgery in the First People’s Hospital of Yongkang from January 2020 to December 2022 were retrospectively reviewed. Among them, 43 patients underwent X-ray guided PFO occlusion surgery (X-ray group) while 47 patients underwent TTE guided PFO occlusion surgery (TTE group). Perioperative, cardiac function related indicators were measured before and after treatment, along with right-to-left shunting status, and incidence of complications in both groups.

**Results:**

There was no significant difference in the duration of surgery or hospitalization between the TTE group and the X-ray group (*p*>0.05). After treatment, the cardiac function indicators of both groups increased compared to before treatment (*p*<0.05), and there was no significant difference between the groups (*p*>0.05). After treatment, right-to-left shunting in the two groups improved compared to before treatment (*p*<0.05), with no significant difference between the groups (*p*>0.05). There was no significant difference in complications between the two groups (*p*>0.05).

**Conclusions:**

TTE guided PFO occlusion is as effective as X-ray guided PFO occlusion in the treatment of PFO. TTE surgery is clinically beneficial for reducing radiation damage with a good safety profile.

## INTRODUCTION

The foramen ovale serves as a pathway for the atrial septum during the fetal period.^1^ This opening can usually close due to increased atrial pressure within one year after birth. If the foramen ovale remains unclosed after the age of three, it is considered patent foramen ovale (PFO).^1^,^2^ PFO can have varying degrees of impact on hemodynamic status.^3^the Risk of Paradoxical Embolism (RoPE Presence of PFO makes right-to-left shunting easier, which may result in the flow of embolic substances from the venous system to the arterial system, thereby increasing the risk of transient ischemic attack, occult stroke and other embolism-related diseases.^3^-^5^ Therefore, early implementation of safe and effective treatment for PFO is important for prevention of these outcomes.

Surgery can be implemented in the treatment of PFO.^6^ Open chest surgery used to be the primary treatment modality, and has been used for lesion repair during a cardiopulmonary bypass.^7^ However, the trauma is significant and the postoperative recovery is slow.^6^,^7^ Percutaneous intervention closure is also commonly used in PFO, as it does not require thoracotomy or extracorporeal circulation.^8^ Traditional treatment is typically guided by X-ray, which can increase the exposure of the operator and patient to radiation, and contrast agents may also cause renal failure.^8^,^9^ Transthoracic echocardiography (TTE) can compensate for the shortcomings of X-ray and clarify the spatial position, morphology, and other information of the inspected site, guiding the implementation of foramen ovale occlusion surgery.^10^

Although there are many studies on the effective of TTE alone, few compare TTE with X-ray guided PFO closure. In the past three years, some patients in our hospital have undergone TTE guided PFO closure surgery. Therefore, this study retrospectively selected patients who underwent PFO closure surgery in our hospital from January 2020 to December 2022 to compare the effectiveness of TTE and X-ray guided PFO closure surgery.

## METHODS

In this retrospective study, clinical data of 90 patients who underwent PFO closure surgery in the First People’s Hospital of Yongkang from January 2020 to December 2022 were retrospectively reviewed. Among them, 43 patients underwent X-ray guided PFO occlusion surgery and were assigned to the X-ray group, while 47 patients received TTE guided PFO occlusion surgery and were assigned to the TTE group.

### Inclusion criteria:

- Patients who comply with PFO diagnostic criteria.^11^
- An age ranges from 18 to 70 years old.
- Transthoracic contrast-enhanced ultrasound examination indicates right-to-left atrial shunt.
- Right atrial catheterization examination indicates a right atrial pressure of ≤ 8 mmHg.
- The clinical data is complete.

### Exclusion criteria:

- There are intracranial organic diseases, such as intracranial stenosis, tumor, or vascular malformation
- Patients with hematologic diseases.
- Factors such as pulmonary hypertension can cause an increase in right atrial pressure, leading to reopening of the foramen ovale.
- Intracardiac thrombosis or infective endocarditis.
- Patients with other heart disease or cardiac malformations requiring open heart surgery.
- Existence of mental diseases.

### Ethical Approval

The Ethics Committee of the First People’s Hospital of Yongkang approved this study on May 29^th^ 2023, No. 202305271710000342703.

### X-ray guided PFO closure surgery^12^

Patients were assisted into a supine position and local anesthesia was given. A puncture sheath was inserted through the right femoral vein, and routine heparinization treatment was administered. Once the patient was ready, right cardiac catheterization was performed. Specifically, the guide wire was completely passed through the foramen ovale and was sent to the left upper pulmonary vein. The guide wire was exchanged and put into the delivery sheath. Under fluoroscopy guidance, the occlude was released along the delivery sheath on both sides of the atrial septum. Once satisfactory sealing was confirmed, the occlude was completely released. The remaining devices were removed and right heart contrast echocardiography was performed again through the elbow vein. Once this was complete, the opening was surgically closed.

### TTE guided PFO closure surgery

The TTE guided PFO closure was completed using Philips EPIQ 7C ultrasound system (Phillips Medical Systems, Andover, MA, USA) X5-1 transducer, frequency 1.0-5.0 MHz.^13^ A single curved catheter was inserted into the left atrium through the foramen ovale, and guided by acoustic contrast examination to confirm that the end catheter was located in the left atrium. The J-shaped hard guide wire was replaced and the catheter was inserted into the delivery sheath. The occluder was inserted into both sides of the atrial septum through the delivery sheath. Once the closure was deemed satisfactory, the occluder was completely released, and the remaining devices were withdrawn. Right heart ultrasound imaging was performed again through the elbow vein, and the opening was surgery closed.

### Observation indicators:

- Perioperative condition.
- Cardiac function related indicators before and after treatment, including left ventricular ejection fraction (LVEF), left ventricular stroke volume (LVSV), and left ventricular end diastolic volume (LVEDV). A Philips IE33 cardiac ultrasound was used to measure the above indicators.
- The presence of a right-to-left shunt before and after treatment was measured and the number of microbubbles in the left atrium were counted and graded as follows: negative, no microbubbles; mild, 1-10 microbubbles; moderate, 11-30 microbubbles; and extensive, ≥30 microbubbles.^13^
- The incidence of complications was observed and recorded for both groups.

### Statistical Analysis

Data was inputted into Microsoft Excel and analyzed using SPSS version 26.0 (IBM Corp, Armonk, NY, USA). Data normality was assessed using the Shapiro Wilk test, and normally distributed data are expressed by mean ± standard deviation. An independent sample t test was used for inter group comparisons, and a paired t test was used for intra group comparisons. Data of non-normally distributed data are expressed as median and interquartile intervals. The Mann Whitney U test was used for inter group comparisons, and the Wilcoxon signed rank test was used for intra group comparisons. The counting data were described as frequency and composition ratio (%), and a Chi-squared test or Fisher’s exact test was performed. The rank sum test was used for rank data. Statistical significance was set at *p*<0.05.

## RESULTS

A total of 90 patients who underwent PFO closure surgery were included in this study. Among them, there were 43 males and 47 females, with an age range from 34 to 69 years old and an average age of 49.0 ± 8.5 years old. The range in BMI was 17.8 to 29.1 kg/m^2^, with an average of 23.7 ± 2.8 kg/m^2^. The diameter of PFO was 0.5 to 1.3 cm, with an average of 0.87 ± 0.19 cm. There was no significant difference in baseline data, surgical duration, or hospitalization duration between the two groups (*p*>0.05) (Table-I). There was no significant difference in cardiac function indicators between the two groups before or after treatment (*p*>0.05). However, after treatment, LVEF, LVSV, and LVEDV in both groups increased compared to before treatment (*p*<0.05) (Table-II). Before and after treatment, there was no significant difference in right-to-left shunting between the two groups (*p*>0.05). After treatment, right-to-left shunting in both groups improved compared to before treatment (*p*<0.05) (Table-III). There was no significant difference in complications between the two groups (*p*>0.05) (Table-IV).

**Table-I:** Comparison of baseline data and perioperative indicators between the two groups.

Information	TTE group (n=47)	X-ray group (n=43)	χ^2^/t/Z	p-Value
Male, n (%)	25 (53.2)	18 (41.9)	1.156	0.282
Age (years)	48.6±8.1	49.4±9.0	-0.420	0.675
BMI (kg/m^2^)	23.4±2.4	23.9±3.2	-0.823	0.413
PFO diameter (cm)	0.87±0.19	0.88±0.18	-0.338	0.736
Surgical duration (min)	38.5±5.2	36.5±6.4	1.613	0.110
Hospitalization duration (days)	1 (1, 1)	1 (1, 1)	-1.344	0.179

**Table-II:** Comparison of cardiac function indicators between two groups.

Time	Group	n	LVEF (%)	LVSV (ml)	LVEDV (ml)
Before treatment	TTE group	47	60.1±5.1	34.6±4.8	58.8±5.2
	X-ray group	43	58.8±5.5	34.1±5.7	57.2±6.7
	t		1.205	0.493	1.290
	p-Value		0.232	0.623	0.200
After treatment	TTE group	47	67.5±3.8^a^	41.7±4.7^a^	67.0±5.9^a^
	X-ray group	43	69.0±4.1^a^	40.1±5.4^a^	65.0±6.9^a^
	t		-1.802	1.563	1.463
	p-Value		0.075	0.122	0.147

***Note:*** Compared with before treatment in this group,

a *p*<0.05.

**Table-III:** Comparison of right-to-left shunting between the two groups.

Time	Group	n	Negative	Mild	Moderate	Extensive
Before treatment	TTE group	47	0 (0.00)	1 (2.13)	2 (4.26)	44 (93.62)
	X-ray group	43	0 (0.00)	2 (4.65)	3 (6.98)	38 (88.37)
	Fisher’s		0.796			
	p-Value		0.627			
After treatment	TTE group	47	42 (89.36)^a^	2 (4.26)^a^	2 (4.26)^a^	1 (2.13)^a^
	X-ray group	43	37 (86.05)^a^	3 (6.98)	2 (4.65)^a^	1 (2.33)^a^
	χ^2^		0.339			
	p-Value		0.952			

***Note:*** Compared with before treatment in this group,

a *p*<0.05.

**Table-IV:** Comparison of the incidence of complications between the two groups.

Group	n	Myocardial infarct	Atrial fibrillation	Severe residual shunt	Severe atrioventricular block	Total occurrence rate
TTE group	47	1 (2.13)	1 (2.13)	2 (4.26)	1 (2.13)	5 (10.64)
X-ray group	43	0 (0.00)	1 (2.33)	1 (2.33)	1 (2.33)	3 (6.98)
χ^2^						0.057
p-Value						0.811

## DISCUSSION

This study retrospectively analyzed the clinical data of patients with PFO who underwent TTE and X-ray guided PFO closure surgery, respectively. There was no significant difference in the surgical duration and hospitalization duration between the TTE group and the X-ray group. Additionally, cardiac function and right-to-left shunting improved in both groups after treatment and there was no significant difference between the two groups. These results highlight that both TTE and X-ray guided PFO occlusion have the same effect in patients with PFO.^8^-^10^ Both surgical treatments can improve patients’ cardiac function and blood flow status, but TTE guided PFO occlusion may be considered more safe due to the lack of X-ray exposure.^9^,^10^,^14^-^16^

TTE guided PFO can avoid X-ray exposure for both the operator and patient, this surgical method does not require large equipment such as DSA, and also has no special requirements for the treatment site.^17^ At the same time, the use of TTE during PFO occlusion can clearly locate the specific position of the foramen ovale, and detect whether the occluder has fallen off or shifted. Additionally, TTE can provide a clearer representation of PFO size and shunt status, which allows more accurate images, personalized treatment, and provides a reference for the development of the best treatment plan.^18^

While TTE guided PFO closure is of high clinical value, this technique requires close cooperation between cardiac ultrasound doctors and PFO closure operators, who are trained in the surgical treatment process. These medical professionals who have rich experience in extracorporeal circulation surgery, cardiac anatomy, or interventional surgery for congenital heart defect, are more likely to master the operation process and skills of PFO closure. Patience, precision, calmness, and composure should be used during treatment to improve the success rate of treatment and reduce the risk of complications.^19^

Unfortunately, the current lack of dedicated end hole catheters for PFO conditions in clinical practice can increase the difficulty of surgical treatment, however, adopting appropriate specifications of end hole catheters during surgery to explore the foramen ovale can significantly improve the success rate of treatment.^20^,^21^ In addition, it is difficult to guide TTE when establishing a track to deliver the occluder sheath. During treatment, it is necessary for the surgeon to evaluate the position of the sheath tip by referring to the friction between the J-type hard guide wire tip and the sheath tip, or to measure the working distance to evaluate the depth of the sheath, in order to ensure the effectiveness and safety of treatment.^22^,^23^

### Limitations

This was a single center, retrospective analysis, with a small sample size. The results of this study only evaluated the right side of the heart using contrast echocardiography immediately after occlusion, without long-term patient follow-up. There was no patient prognosis data collected or included in analysis. Further trials utilizing a larger sample size, long-term follow-up or randomized patient allocation are required to verify the results of this study.

## CONCLUSION

TTE guided PFO occlusion is as effective as X-ray guided PFO occlusion in the treatment of PFO. TTE surgery is clinically beneficial for reducing radiation damage with a good safety profile.

### Authors’ contributions:

**HS:** Conceived and designed the study.

**HS** and **YY:** Collected the data and performed the analysis.

**HS:** Was involved in the writing of the manuscript and is responsible for the integrity of the study.

All authors have read and approved the final manuscript.

## References

[1] Elzanaty AM, Patel N, Sabbagh E, Eltahawy EA (2021). Patent foramen ovale closure in the management of cryptogenic stroke:a review of current literature and guideline statements. Curr Med Res Opin.

[2] Kent DM, Saver JL, Kasner SE, Nelson J, Carroll JD, Chatellier G (2021). Heterogeneity of Treatment Effects in an Analysis of Pooled Individual Patient Data From Randomized Trials of Device Closure of Patent Foramen Ovale After Stroke. JAMA.

[3] Kent DM, Saver JL, Ruthazer R, Furlan AJ, Reisman M, Carroll JD (2020). Risk of Paradoxical Embolism (RoPE)-Estimated Attributable Fraction Correlates With the Benefit of Patent Foramen Ovale Closure:An Analysis of 3 Trials. Stroke.

[4] Ali SR, Ahmed S, Aslam N, Lohana H (2016). Atrial Thrombus in a Premature Newborn Following Cardio-Pulmonary Resuscitation. J Coll Physicians Surg Pak.

[5] Gonzalez JB, Testai FD (2021). Advances and Ongoing Controversies in Patent Foramen Ovale Closure and Cryptogenic Stroke. Neurol Clin.

[6] Aftanski P, Maloku A, Dannberg G, Hamadanchi A, Günther A, Schulze PC (2022). Closure of a Patent Foramen Ovale (PFO):An Intervention Sequence. J Vis Exp.

[7] Farjat-Pasos JI, Guedeney P, Houde C, Alperi A, Mesnier J, Côté M (2022). Transcatheter Closure of Patent Foramen Ovale in Patients With Peripheral (Noncerebrovascular) Embolism. J Invasive Cardiol.

[8] Nishtar T, Ullah N, Ahmad FS, Rahim S (2022). Radiographic patterns on Chest X-ray as a supporting imaging tool in triaging of suspected Corona Virus Disease (COVID) patients. Pak J Med Sci.

[9] Rehman K, Mustafa G, Khan MA, Rauf Z, Khan N, Khan S (2019). Clinical Biomarkers for Diagnosis of Damages in Individuals with Long-Term Exposure to X-Rays. J Coll Physicians Surg Pak.

[10] Akagi T (2021). Transcatheter closure of patent foramen ovale:Current evidence and future perspectives. J Cardiol.

[11] Ntaios G, Tzikas A, Vavouranakis E, Nikas D, Katsimagklis G, Koroboki E (2020). Expert consensus statement for the management of patients with embolic stroke of undetermined source and patent foramen ovale:A clinical guide by the working group for stroke of the Hellenic Society of Cardiology and the Hellenic Stroke Organization. Hellenic J Cardiol.

[12] Achim A, Hochegger P, Kanoun Schnur SS, Moser L, Stark C, Pranevicius R (2023). Transesophageal echocardiography-guided versus fluoroscopy-guided patent foramen ovale closure:A single center registry. Echocardiography.

[13] Tian L, Zhang M, Nie H, Zhang G, Luo X, Yuan H (2023). Contrast-enhanced transcranial doppler versus contrast transthoracic echocardiography for right-to-left shunt diagnosis. J Clin Monit Comput.

[14] Tang S, Zhou Y, Liu L, Zhao F, He J, Zheng Bo (2022). Analysis of the safety and clinical value of percutaneous closure of patent foramen ovale guided by simple transthoracic echocardiography. Sichuan Medicine.

[15] Yang T, Ouyang W, Zhao G, Zhang F, Pan X (2019). Safety and Efficacy of Percutaneous Closure of Patent Foramen Ovale Guided Solely by Transthoracic Echocardiograph. Chinese Circul J.

[16] Ates AH, Yorgun H, Canpolat U, Sener YZ, Oksul M, Kaya EB (2021). Long-term follow-up outcomes in a real-world study cohort after percutaneous patent foramen ovale closure. Turk Kardiyol Dern Ars.

[17] Arfaras-Melainis A, Palaiodimos L, Mojadidi MK (2019). Transcatheter Closure of Patent Foramen Ovale:Randomized Trial Update. Interv Cardiol Clin.

[18] Poloczek M, Kala P, Ondrúš T (2021). Patent foramen ovale from the point of view of interventional cardiology. Vnitr Lek.

[19] Messé SR, Gronseth GS, Kent DM, Kizer JR, Homma S, Rosterman L (2020). Practice advisory update summary:Patent foramen ovale and secondary stroke prevention:Report of the Guideline Subcommittee of the American Academy of Neurology. Neurology.

[20] Donti A, Egidy Assenza G, Mariucci E (2019). [Transcatheter closure of patent foramen ovale in patients with cryptogenic stroke]. G Ital Cardiol (Rome).

[21] Yang T, Butera G, Ou-Yang WB, Zhao GZ, Zhang FW, Pan XB (2019). Percutaneous closure of patent foramen ovale under transthoracic echocardiography guidance-midterm results. J Thorac Dis.

[22] Alushi B, Lauten A, Cassese S, Colleran R, Schüpke S, Rai H (2018). Patent foramen ovale closure versus medical therapy for prevention of recurrent cryptogenic embolism:updated meta-analysis of randomized clinical trials. Clin Res Cardiol.

[23] Vitarelli A (2019). Patent Foramen Ovale:Pivotal Role of Transesophageal Echocardiography in the Indications for Closure, Assessment of Varying Anatomies and Post-procedure Follow-up. Ultrasound Med Biol.

